# Supplementary material for: All-at-once RNA folding with 3D motif prediction framed by evolutionary information
Source: Nat Methods. 2025 Oct 3;22(10):2094–106. doi: 10.1038/s41592-025-02833-w (PMC12510878; doi:10.1038/s41592-025-02833-w)
Supplement: Supplementary file 3 — Source code to run CaCoFold-R3D. Data and results. [file 41592_2025_2833_MOESM3_ESM.gz › supplemental_material/Figures/Figure_S4.pdf]

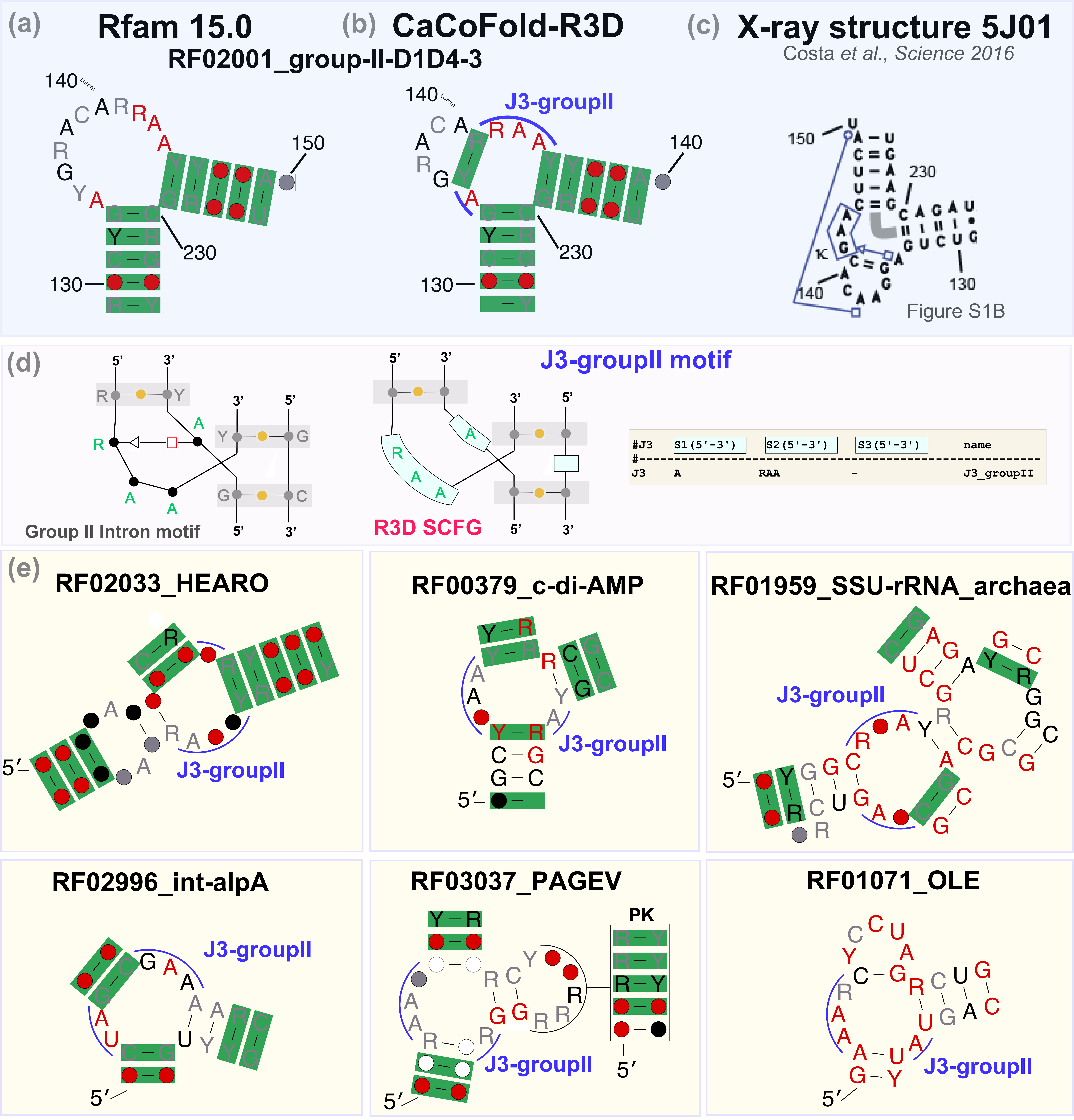
(a) Rfam 15.0
RF02001_group-II-D1D4-3
(b) CaCoFold-R3D
J3-groupII
(c) X-ray structure 5J01
Costa et al., Science 2016
Figure S1B
(d)
Group II Intron motif
R3D SCFG
J3-groupII motif
#J3 S1(5'-3') S2(5'-3') S3(5'-3') name
J3 A RAA - J3_groupII
(e) RF02033_HEARO
RF00379_c-di-AMP
RF01959_SSU-rRNA_archaea
RF02996_int-alpA
RF03037_PAGEV
PK
RF01071_OLE
J3-groupII
